# DIA/SWATH-Mass Spectrometry Revealing Melanoma Cell Proteome Transformations with Silver Nanoparticles: An Innovative Comparative Study

**DOI:** 10.3390/ijms26052029

**Published:** 2025-02-26

**Authors:** Simona Martano, Jakub Faktor, Sachin Kote, Mariafrancesca Cascione, Riccardo Di Corato, Dagmar Faktorova, Paola Semeraro, Loris Rizzello, Stefano Leporatti, Rosaria Rinaldi, Valeria De Matteis

**Affiliations:** 1 Department of Mathematics and Physics “Ennio De Giorgi”, University of Salento, Via Arnesano, 73100 Lecce, Italy; simona.martano@unisalento.it (S.M.); mariafrancesca.cascione@unisalento.it (M.C.); ross.rinaldi@unisalento.it (R.R.); 2 International Centre for Cancer Vaccine Science, University of Gdansk, Kladki 24, 80-822 Gdansk, Poland; jakub.faktor@ug.edu.pl; 3 Institute for Microelectronics and Microsystems (IMM), CNR, Via Monteroni, 73100 Lecce, Italy; riccardo.dicorato@cnr.it; 4 Center for Biomolecular Nanotechnologies, Istituto Italiano di Tecnologia (IIT), 73010 Arnesano, Italy; 5 Faculty of Special Technology, Alexander Dubček University of Trenčín, 911 06 Trenčín, Slovakia; dagmar.faktorova@tnuni.sk; 6 Department of Biological and Environmental Sciences and Technologies (DiSTeBA), University of Salento, Via Monteroni, 73100 Lecce, Italy; paola.semeraro@unisalento.it; 7 Department of Pharmaceutical Sciences, University of Milan, 20133 Milan, Italy; loris.rizzello@unimi.it; 8 CNR Nanotec-Istituto Di Nanotecnologia, C/O Campus Ecotekne, Via Monteroni, 73100 Lecce, Italy; stefano.leporatti@nanotec.cnr.it; 9 Department of Experimental Medicine, University of Salento, Via Monteroni, 73100 Lecce, Italy

**Keywords:** green silver nanoparticles, DIA/SWATH-mass spectrometry, melanoma, proteomic profile

## Abstract

Melanoma is an aggressive cancer with rising incidence and high mortality rates, largely due to chemotherapy resistance and molecular dysregulation. Nanotechnology, particularly silver nanoparticles (AgNPs), has emerged as a promising therapeutic avenue because of the nanoparticles’ ability to induce oxidative stress and apoptosis in cancer cells. However, conventional colloidal AgNPs lack selectivity, often causing significant damage to healthy cells. In this study, we introduce a green synthesis of AgNPs using plant extracts, providing an eco-friendly alternative with improved antitumor selectivity compared to traditional colloidal AgNPs. Leveraging label-free Data-Independent Acquisition/Sequential Window Acquisition of All Theoretical Mass Spectrometry (DIA/SWATH MS) quantitative proteomics, we investigated the antitumor effects of green-synthesized versus traditional AgNPs on A375 melanoma cells at 24 and 48 h. Our findings reveal that green AgNPs selectively reduced melanoma cell viability while sparing healthy keratinocytes (HaCaT), a benefit not observed with colloidal AgNPs. Proteomic analysis highlighted that green AgNPs significantly downregulated oncogenes, enhanced carbohydrate metabolism, and disrupted copper homeostasis in melanoma cells. This marks the first study to explore the differential effects of green and traditional AgNPs on melanoma using an integrated proteomic approach, underscoring the molecular potential of green AgNPs as a targeted and sustainable option for cancer therapy.

## 1. Introduction

Melanoma, a multifactorial disorder, is a significant global health issue due to its rising incidence, aggressive nature, and the high costs associated with its prevention, diagnosis, and treatment [1]. Its propensity for advanced metastasis and chemotherapy resistance contributes to high mortality rates [2]. Melanoma incidence has rapidly increased globally, especially in light-skinned populations in regions like North America, Northern Europe, Australia, and New Zealand, with annual rates rising by 4–6%. In 2020, there were approximately 325,000 new cases and 57,000 deaths, making melanoma one of the most common cancers worldwide. Projections indicate a 57% increase in new cases and a 68% increase in deaths by 2040 [1].

At the molecular level, dysregulation of intracellular signaling pathways, driven by autocrine growth factor secretion, mutations in regulatory genes, and loss of adhesion receptors, plays a critical role in melanoma progression [3]. Considering this, the need for therapeutic tools based on a deeper understanding of melanoma pathology has led researchers to explore innovative nanotechnology solutions for this pressing public health issue [4,5]. Malignant melanoma, the most lethal form of skin cancer, shows resistance to drug-induced apoptosis [6]. These proteins prevent the activation of caspases, which are essential for programmed cell death, by inhibiting the mitochondrial and death receptor signaling pathways [7]. The combination of understanding the role of apoptotic regulators, alongside targeting alternative cell death pathways and using novel treatment strategies, could improve the treatment of melanoma and overcome resistance to conventional therapies. Melanoma resistance to targeted therapies involves re-activation of the MAPK pathway and activation of the PI3K-mTOR pathway. The tumor microenvironment, including stromal cells, also plays a key role in supporting resistance. Additionally, melanoma cells upregulate autophagy in response to drug-induced stress, a process driven by ER stress and TAM receptor activation. miRNA-mediated mechanisms further contribute to therapeutic resistance [8].

Nanotechnology offers a range of tools for cancer treatment, particularly metal nanoparticles (NPs) [9]. Among these, silver nanoparticles (AgNPs) stand out for their ability to induce toxicity in cancer cells by altering cellular morphology, reducing cell viability, and triggering oxidative stress [10,11]. Although several physical and chemical synthetics method for AgNPs are discussed in the literature [12,13,14,15], the bio-mediated approach has gained attention due to the demand for eco-friendly (“green”) technologies [16,17,18]. Green synthesis methods use polyphenols from leaf extracts as reducing and stabilizing agents for metallic ions like silver, avoiding toxic and time-consuming approaches. These methods typically produce NPs ranging from 10 to 100 nm [19], which is crucial for their anti-tumor properties. The cytotoxic effects of AgNPs on cancer cells include generating reactive oxygen species (ROS), cell cycle disruption, and genotoxic impacts, leading to inflammation, apoptosis, and cell death [15,20,21,22]. Additionally, specific protein expression can be altered [23,24].

Here, we present a comprehensive label free Data-Independent Acquisition (DIA)/Sequential Window Acquisition of All Theoretical Fragment Ion Spectra (SWATH) quantitative proteomics analysis for determining the global proteomic changes in the human A375 melanoma cell line upon exposure to AgNPs synthesized via conventional and green routes at two time points (24 and 48 h). The selected time points, commonly used in nanoparticle studies, enable the assessment of cellular responses over short- and mid-term exposure periods, capturing both early molecular changes and cellular adaptations. This approach also aligns with previous studies, ensuring comparability and relevance within the field. Together, DIA/SWATH is a quantitative proteomic mass spectrometry method combining deep proteome coverage with accuracy and consistency. Following an LC-MS method described in our previous publication [25], SWATH/DIA MS acquisition measures all ionized peptides in each sample that fall within a specified *m*/*z* precursor range by splitting it into isolation windows. The precursors in each window are simultaneously fragmented, and their spectra are recorded consecutively [26]. By doing so, thousands of proteins could be reproducibly quantified in across multiple samples, making this process an appropriate tool for up-to-date proteomic research.

Prior to cell experiments, AgNPs were fully characterized by Transmission Electron Microscopy (TEM), Dynamic Light Scattering (DLS), and zeta potential. Raman Spectroscopy was used to assess the organic vibration related to organic compounds on the surface. This study seeks to unveil the precise proteins and cellular networks influenced by AgNPs treatments, highlighting key shifts in the proteomic landscape of human melanoma cells. To the best of our knowledge, this is the first study to investigate the effects of differently synthesized AgNPs on melanoma cells using an integrated DIA/SWATH-MS quantitative proteomics approach.

## 2. Results

By utilizing eco-friendly AgNPs derived from *Laurus nobilis* leaf extract and colloidal AgNPs, our results indicate that AgNPs exposure impairs gene activity, decreasing cell viability in cancer cells while preserving healthy HaCaT keratinocytes. Proteomic analysis revealed significant changes indicative of the activation or suppression of key biological pathways. Notably, the proteins altered in response to AgNPs treatment were linked to critical processes such as the cell cycle, cell morphology, cellular functions, endoplasmic reticulum stress, oxidative stress, and mitochondrial dysfunction, ultimately leading to cell death through apoptosis and/or autophagy activation. Specifically, green AgNPs induced dysregulation of key proteins involved in essential pathways, resulting in the inhibition of oncogene expression, modulation of carbohydrate metabolism, and disruption of copper homeostasis.

### 2.1. Physicochemical Characterization of AgNPs

#### 2.1.1. Morphology and Size Analysis

**Figure 1:**
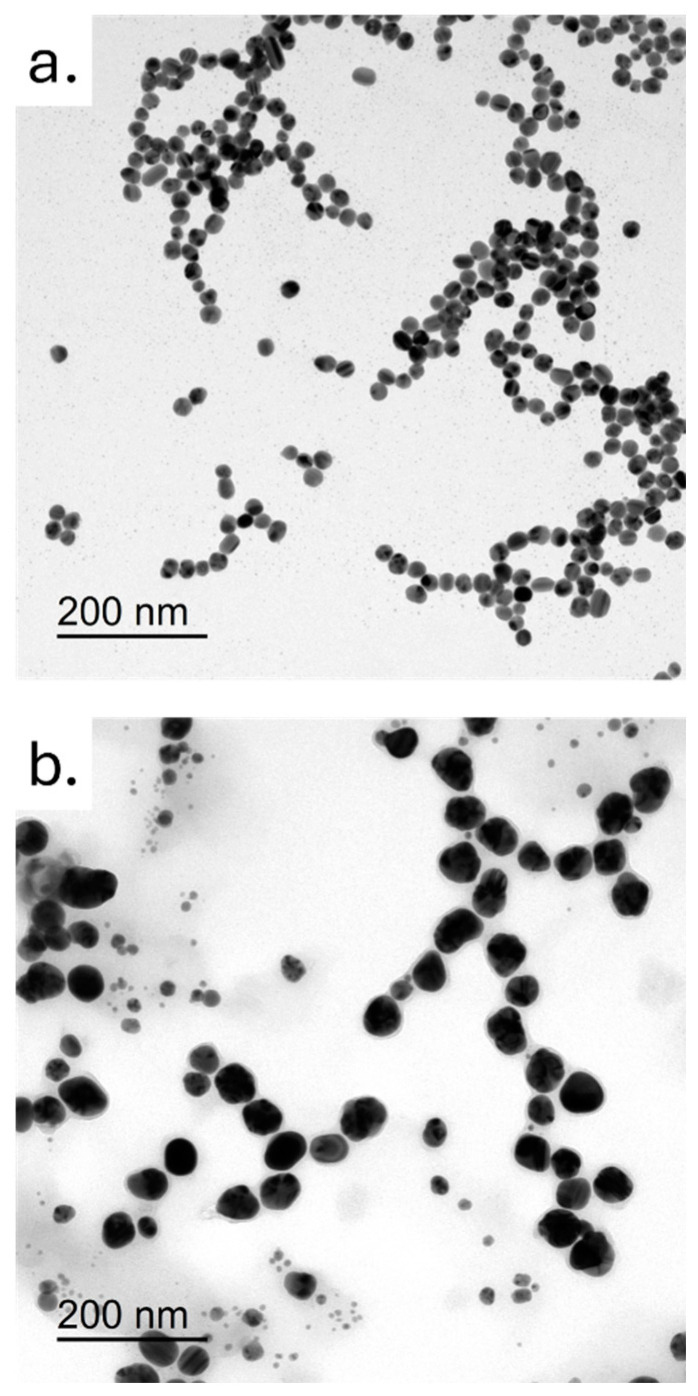
Representative TEM images of colloidal (**a**) AgNPs and (**b**) green AgNPs. Scale bar was 200 nm.

The morphology and size of the two types of AgNPs were analyzed by TEM (Figure 1a,b). Colloidal AgNPs had a size of (20 ± 3) nm (Figure 1a), whereas the green AgNPs were bigger, showing a diameter of (35 ± 7) nm (Figure 1b). Shape was nearly comparable between the AgNPs derived from the two synthetic routes, confirming an almost spherical morphology, although it was more defined in the case of colloidal AgNPs. Overall, these nanostructures appeared to be generally monodispersed, and slightly tended to aggregate into small clusters.

#### 2.1.2. Hydrodynamic Size and Stability in Cell Culture Medium

The size of the AgNPs was further confirmed through DLS analysis. DLS measurements conducted in water provided evidence of compatibility between the measured hydrodynamic radius of the AgNPs and the average size values detected in the TEM acquisitions. In particular, the hydrodynamic radius recorded for the green and colloidal AgNPs is (36 ± 8) nm and (21 ± 3) nm, respectively. These measurements align closely with the TEM analysis. To evaluate the stability of AgNPs in cell culture medium, DLS measurements were performed in DMEM. The peculiar formation of the protein corona on the NP surface led to an enhanced size value of (38 ± 7) nm for green AgNPs and (28 ± 3) for the colloidal ones (Table 1).

#### 2.1.3. Surface Charge and Zeta Potential

Moreover, assuming the dual functionality of polyphenols extracted from plants as both natural reducing and capping agents, the surface charge was found to be negative (−30 ± 6 mV) and (−23 ± 2 mV) for green and colloidal AgNPs, respectively. Furthermore, the ζ-potential underwent a change due to the significant presence of serum proteins on the surface, becoming more negative (−41 ± 6 mV) and (−32 ± 5 mV). Additionally, an observed shift in the ζ-potential was noted, likely attributed to the substantial abundance of serum proteins on the surface, resulting in a more negative charge of (−41 ± 6 mV) (Table 1).

#### 2.1.4. Optical Properties: UV-Vis Analysis and Raman Spectroscopy

**Figure 2:**
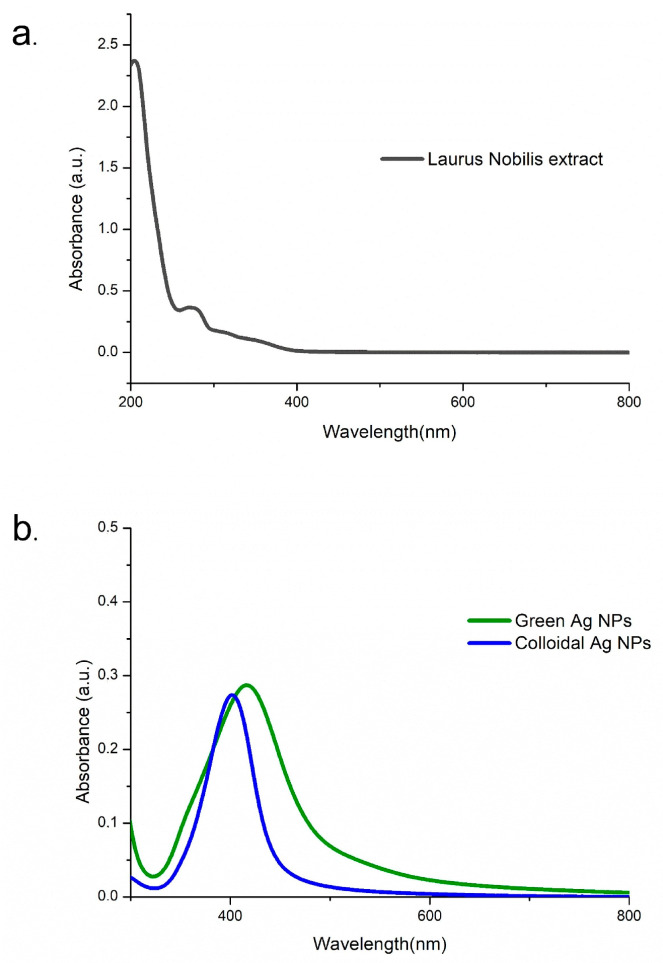
UV-Vis spectra of the samples: (**a**) *Laurus nobilis* extract; (**b**) green AgNPs and colloidal AgNPs.

The UV-Vis absorption spectra of NPs were recorded in the range of 300–800 nm and compared with the spectrum of the corresponding *Laurus nobilis* leaf extract (Figure 2). The analysis was conducted using the leaf extract, revealing the presence of a peak in the UV region at around 280 nm, likely stemming from the presence of aromatic compounds. Within the wavelength range of 300–800 nm, no absorption signal was detected. The spectrum of AgNPs obtained through the green approach showed a slightly broader peak compared to the peak of colloidal AgNPs, which was perfectly centered at 400 nm (typical value of absorption for spherical AgNPs).

**Figure 3:**
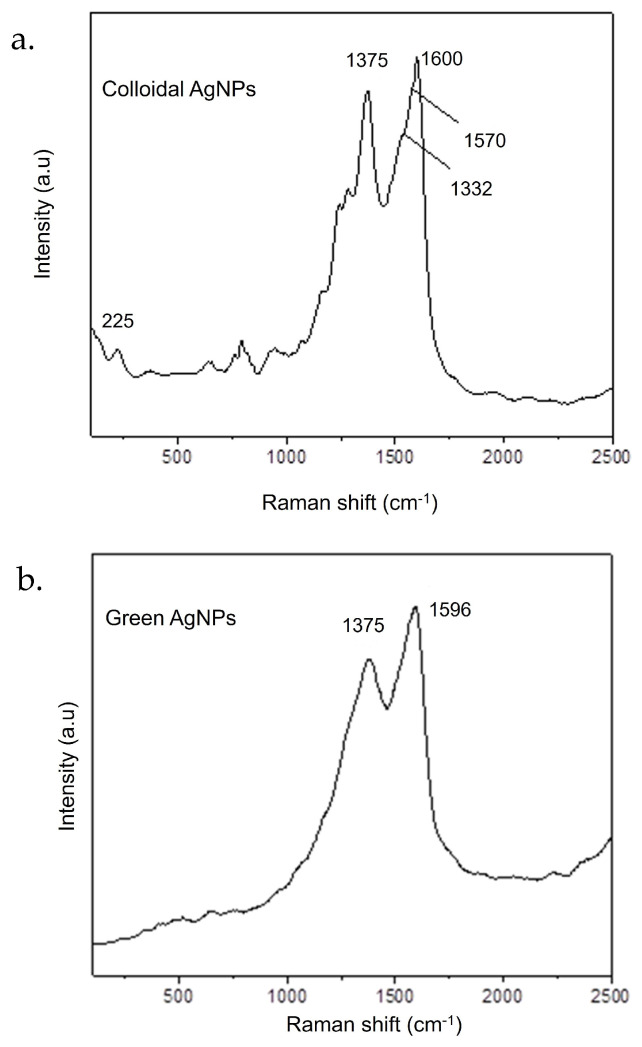
Raman spectra of colloidal AgNPs (**a**) and green AgNPs (**b**).

The Raman spectrum of colloidal AgNPs (Figure 3a) shows several weak and shouldered signals ascribed to the citrate used as a reducing agent in nanoparticle synthesis. The weak band at 225 cm^−1^ could be assigned to the Ag–O stretching mode, which indicates the formation of a chemical bond between silver and carboxylate groups of citrate molecules [27]. The low and wider signal at about 660 cm^−1^ and the bands between 756-842 cm^−1^ could be assigned to the COO bending mode, while the shoulder at 1575 cm^−1^ could instead be ascribed to symmetric stretching vibrations of COO [28]. The intense and sharp signals at 1372 and 1600 cm^−1^ could be attributed to the vibrational mode of C=O [29]. On the contrary, the Raman spectrum of green AgNPs (Figure 3b) showed only two intense and broad peaks at about 1375 and 1596 cm^−1^ due to the overlapping of different bands, which could be attributed to the vibrational mode of C=O or to symmetric stretching vibrations of COO^−^ and C–C stretching vibrations of aromatic ring [27,29] present in the molecular structure of polyphenols.

### 2.2. Biological Assessment of AgNPs

#### 2.2.1. Cytotoxicity in A375 Melanoma Cells

**Figure 4:**
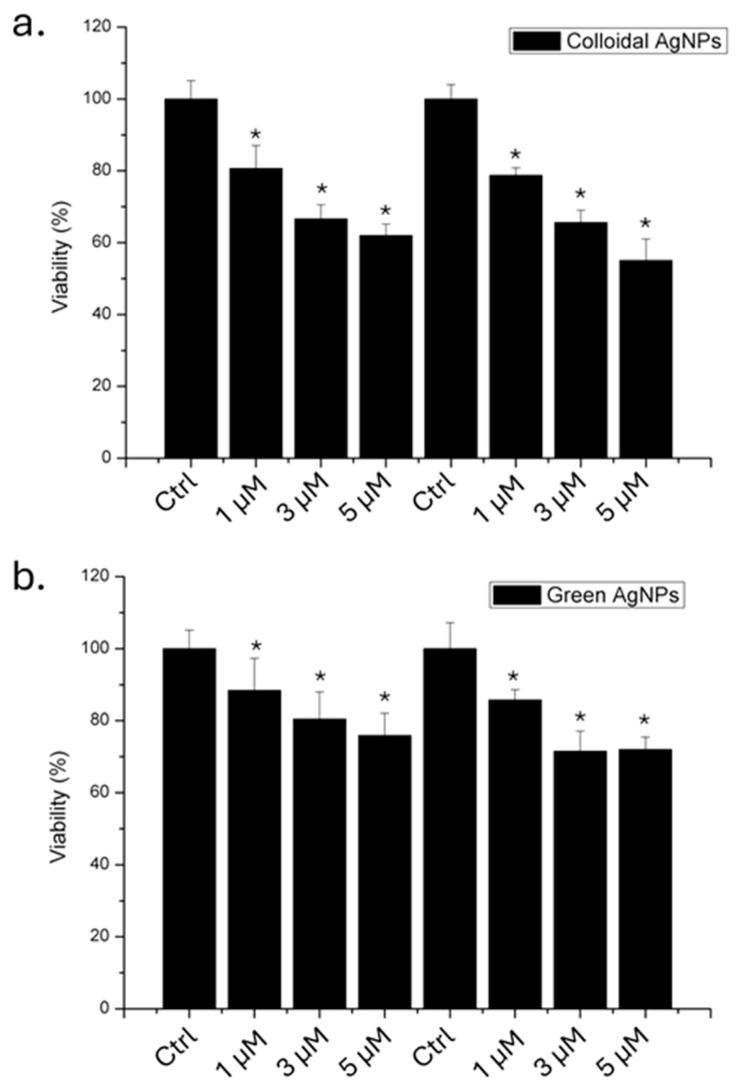
Viability assay (WST-8) of A375 cell lines exposed to 1 µM, 3 µM, and 5 µM of colloidal (**a**) and green (**b**) AgNPs. The viability of NP-treated cells was normalized to non-treated control cells. As a positive control (P), the cells were incubated with 5% DMSO. Data reported as the mean ± SD from three independent experiments are considered statistically significant, compared with the control (*n* = 8) for *p* value < 0.05 (<0.05 *).

Following the physico-chemical characterization of both types of AgNPs, we evaluated their impact on A375 cells in terms of viability using 1, 3, and 5 µM of AgNPs, for 24 and 48 h. The results obtained through WST-8 assay (Figure 4) showed that the colloidal AgNPs were more toxic than green AgNPs. We demonstrated that, after 48 h using the higher concentration, 53% of the cells remained viable. In contrast, when the cells were treated with green AgNPs, 78% of the cells were vital. Thus, the anticancer effect was visible for both types of NPs, even if the effect was stronger for colloidal AgNPs in a time and concentration dependent manner.

#### 2.2.2. Cytotoxicity in HaCaT Keratinocytes

**Figure 5:**
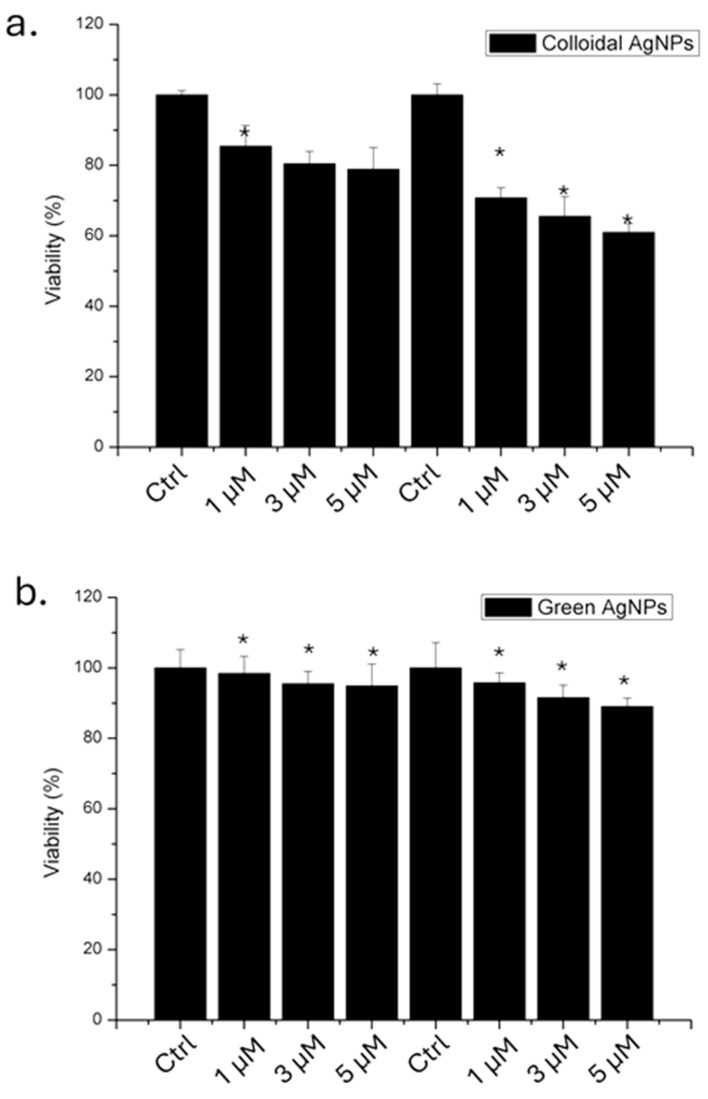
Viability assay (WST-8) of HaCaT cell lines exposed to 1 µM, 3 µM, and 5 µM of colloidal (**a**) and green (**b**) AgNPs. The viability of NP-treated cells was normalized to non-treated control cells. As a positive control (P), the cells were incubated with 5% DMSO. Data reported as the mean ± SD from three independent experiments are considered statistically significant, compared with the control (*n* = 8) for *p* value < 0.05 (<0.05 *).

To assess the impact on healthy cell lines, a viability assay was also conducted on HaCaT cells, which serve as the non-tumoral counterpart to melanoma cells (Figure 5).

The results demonstrated that green AgNPs did not induce a significant reduction in viability compared to colloidal AgNPs. In detail, colloidal AgNPs at a concentration of 5 µM induced a 40% reduction in cell viability after 48 h. Conversely, cells exposed to green AgNPs at the same concentration remained 88% viable. This finding is important in the context of using this type of AgNP as an antitumor agent.

To determine whether there were differences in uptake between the two cell lines, silver concentration was measured after incubating HaCaT and A375 cells with the higher concentration used for viability tests for 48 h. The results show that there were no substantial differences in uptake between the two cell lines, regardless of the type of AgNPs used. This indicates that the selective cytotoxic effect of the green AgNPs on melanoma cells is indeed due to their surface chemical composition, which, in contrast, spares non-tumorigenic cells from mortality. The data obtained from ICP analysis are reported in Table 2.

### 2.3. Mass Spectrometry Benchmark of the Effect of Green AgNPs and Colloidal AgNPs on Proteotype of A375 Melanoma Cells

#### 2.3.1. Mass Spectrometry Approach and Experimental Setup

To elucidate the molecular mechanisms driving the selective properties of green AgNPs over melanoma cells, we implemented DIA/SWATH quantitative mass spectrometry. This is a well-established method for comparing proteotypes or evaluating the impact of treatment on protein level. In our mass spectrometry screen, we compared the proteotypes of both green AgNPs and colloidal AgNPs treated cells harvested at two timepoints, 24 h and 48 h after treatment. Three biological replicates measured in a technical duplicate yielding six MS runs per condition were acquired. Initially, we examined the variability in our mass spectrometry data by evaluating the protein intensities, proving relative consistency of the dataset with only minor effects of sample preparation and/or mass spectrometry contributing to variability. Particularly, we detected a slightly greater extent of 0 or N/A values in two technical replicates of GreenAgNPs_48h, one replicate of WTCellsAgNPs_24h, and one replicate of WTCellsAgNPs_48h (Supplementary Figure S1, protein intensity QC plot).

#### 2.3.2. PCA Analysis of Proteotype Variability

**Figure 6:**
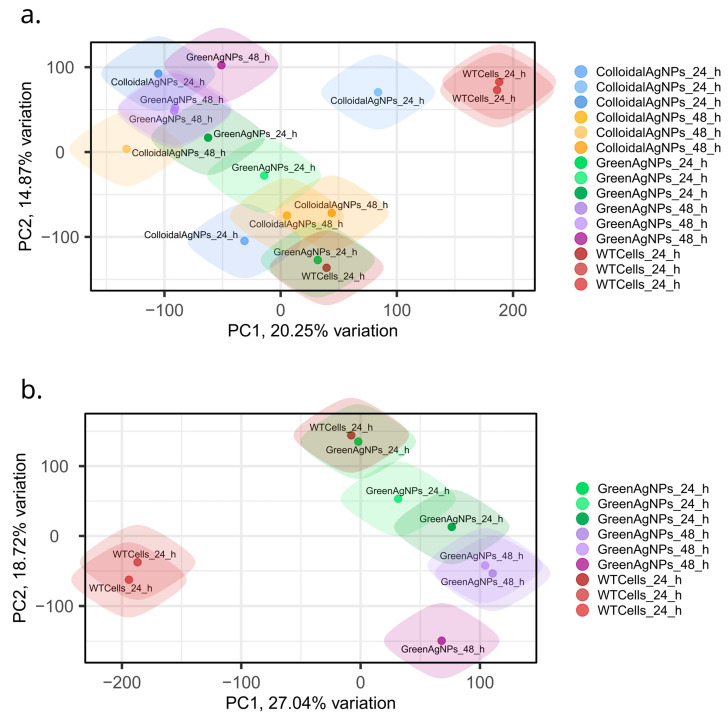
The principal component analysis (PCA) plot analysis. (**a**) PCA of both colloidal and green AgNPs. (**b**) PCA of green AgNPs most promising therapy candidate. PCA indicates clear separation of control cells (WT) and green AgNPs treated melanoma cells after 24 h treatment, which further developed over the next 48 h of treatment; apparently, this observation is reflected in PC1 component.

To further determine variability among induced proteotypes, we performed two PCA analyses (Figure 6a,b). Initially, proteotypes of colloidal and green AgNPs in both timepoints were investigated using principal component analysis (PCA). PCA analysis in Figure 6a highlights that the A375 cells treated with green AgNPs or colloidal AgNPs are exhibiting almost distinct protein intensity profiles compared to control/wild type counterparts (WT) as there is a visible trend in separation along PC1, reflecting relative proteotype dissimilarity. However, a deeper insight into the global differences of green AgNPs and WT proteotypes (Figure 6b) provides even clearer separation, particularly of control cells and green AgNPs treated melanoma cells. Proteotypes of WT and green AgNPs treated cells (both at 24 and 48 h) profile are positioned farther apart, displaying higher proteotype dissimilarity. This observation is in concert with viability assay, if we extrapolate it to the selective green AgNPs effect over A375 cells. In addition, the PCA in Figure 6b shows temporal classification of the proteotypes along PC2, distinguishing the timepoints of green AgNPs treatment of A375 cells.

Nevertheless, we consider these observations, which rely on global proteome changes, to be imprecise but promising, as the separation along PC1 and PC2 might be driven by the proteins related to the selective mechanism of green AgNPs acting over melanoma A375 cells. Therefore, we further thoroughly examined the expression values of discrete proteins across conditions.

#### 2.3.3. Differential Proteome Response and Key Molecular Insights

**Figure 7:**
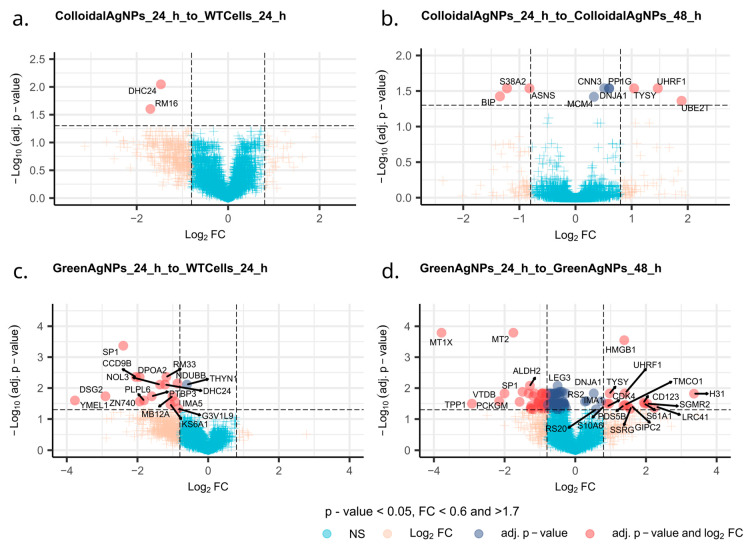
V-plots of the comparisons: (**a**) colloidal AgNPs 24 h to WT cells 24 h; (**b**) colloidal AgNPs 24 h to colloidal AgNPs 48 h; (**c**) Green AgNPs 24 h to WT Cells 24 h; (**d**) Green AgNPs 24 h to green AgNPs 48 h.

Delving into the changes of discrete proteins (Supplementary Table S1, DIA Quantitation results from all comparisons) and comparing them as a function of time in both green and colloidal AgNPs to the WT cells reveals a remarkable difference in responses to the treatments on proteome level, which are apparently in concert with observations from cellular viability assay and from PCA. Assuming that the effect of the decrease in the A375 cell viability will be reflected into dysregulation of proteins, we see a larger pool of dysregulated proteins (Figure 7c,d) upon green AgNPs treatment at any point if compared to colloidal AgNPs (Figure 7a,b), respectively.

To uncover the molecular essence of more potent green AgNPs action, we thoroughly inspected the protein roles and their involvement in key biochemical processes. The exposure of melanoma cells to green AgNPs for 24 h predominantly resulted in the downregulation of cellular proliferation, survival, and differentiation compared to WT cells (Please refer to Section 3). This reflects the cellular response primarily to DNA damage, programmed cell death, and autophagy. Moreover, proteins involved in motility, mitochondrial protein transport and stress response, DNA repair, autophagy, cell cycle regulation, and chromatin organization were further decreased over the next 24 h of green AgNPs action (Figure 7c,d). Knowingly, many of revealed processes might be involved in an antioncogenic effect; however, some these processes have already been described as a general effect of NP treatment. In addition, some of the proteins from which we inferred the shift in processes/pathways might be involved in multiple processes; thus, the interpretation of the meaning of change in the protein level this way might not be accurate.

### 2.4. Focusing on Revealing the Anti-Oncogenic Effect of Green AgNPs Using DIA/SWATH Mass Spectrometry Data

**Figure 8:**
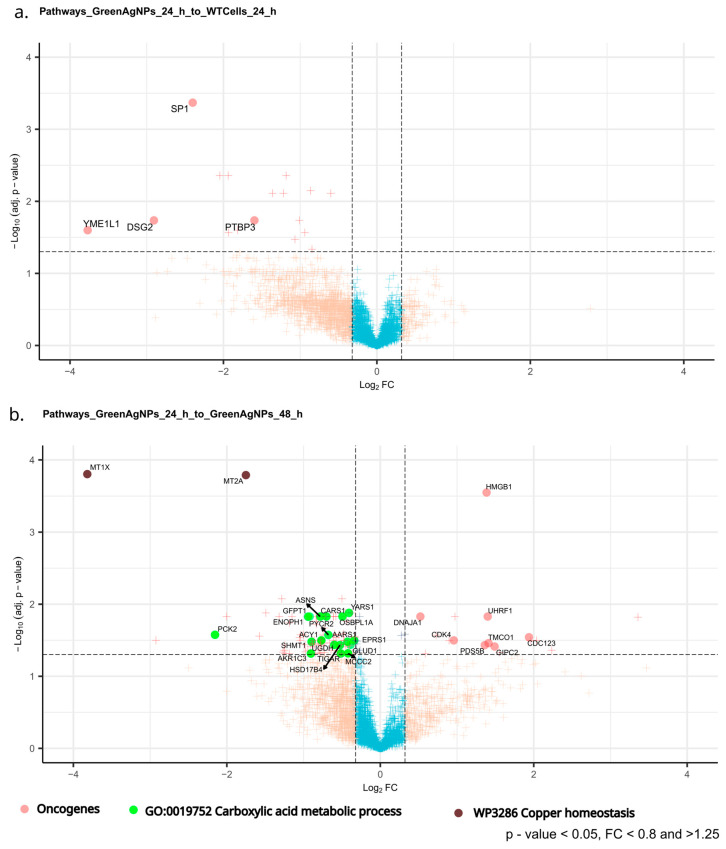
V-plot shows the most profound biological mechanism/proteomics markers expression upon the treatment of green AgNPs. (**a**) Known oncoproteins decrease after incubation with green AgNPs in both time points. (**b**) While there is an increase in metabolism of carboxylic acids after longer incubations (48 h) with particles, which might indicate an increase in normal carbohydrate metabolism via the Krebs cycle (and oxidative phosphorylation) rather than anaerobic glycolysis, which is well known for the cancer state (Warburg effect). This subtle shift might further prove the healing effect of the particle. Dysregulation in genes involved in copper homeostasis might reflect the reaction of the cells to silver.

We further examined the dysregulated protein pool in green AgNPs treated A375 cells, focusing on subtle shifts in processes/pathways involving multiple proteins (Gene ontology—GO) and providing rather cumulative evidence of green AgNPs antioncogenic effect/s (Figure 8a,b).

The most notable effects of green AgNPs on A375 melanoma cells could be represented by key proteins that were either down- or upregulated upon green AgNPs treatment, and that are linked by GO-terms or common function providing cumulative evidence in their role, such as driving oncogenic transformation/processes (Figure 8a,b, Table 3). In addition, our dataset allows us to screen the evolution of this effect in a time-sensitive manner, i.e., in two timepoints (Figure 8a,b, Table 2). Initially, exposure to green AgNPs (24 h) reveals (Figure 8a and Table 3) a downregulation of several oncogenes known for their involvement in the tumorigenesis, cell proliferation, cell invasion, and metastasis: PTBP3, SP1, DSG2, and YME1L.

Extending the incubation time of melanoma cells with nanoparticles to 48 h (Figure 8b, Table 3), which can lead to even more significant decrease in the expression level and number of oncoproteins, clearly amplifies the cancer cell growth inhibition as a function of the treatment time. Also peculiar is the profound onset of carboxylic acids in the metabolism after longer incubations (48 h) with nanoparticles, which might indicate an increase in aerobic glycolysis and Krebs cycle rather than cancer-related anaerobic glycolysis, which is a prominent feature of cancer cells’ metabolism (Warburg effect). In addition, as clearly shown in the volcano plot (Figure 8b, Table 3), the presence of dysregulated genes required in copper homeostasis (MT1X, MT2A) might reflect the cancer cell’s response to AgNPs. On the other hand, copper is a crucial element in all solid tumors for the induction of angiogenesis, which is fundamental for the supply of oxygen and nutrients to the tumor [30]. However, this point needs further clarifications.

## 3. Discussion

The synthesis of metal NPs, including silver ones, through green chemistry starting from plant extracts represents an innovative and eco-friendly strategy compared to conventional colloidal synthesis techniques, thus emerging as a valid alternative for the design of promising nanomaterials appliable in nanomedicine [18,31,32]. Based on this, we utilized laurel leaves as the green raw material in our eco-friendly synthesis approach. In this method, the natural polyphenols present in the extract facilitate the production of stable AgNPs in an aqueous solution, eliminating the need for conventional toxic capping or reducing agents. Both synthesis methods—the traditional colloidal and the green approach—proved to be reproducible, yielding highly concentrated NP solutions. These results align with findings from previous studies [19,33]. AgNPs obtained from the two synthetic routes have been extensively characterized from a physical and chemical point of view, using Transmission Electron Microscopy (TEM), Dynamic Light Scattering (DLS), Z-potential analysis, Inductively Coupled Plasma Atomic Emission Spectroscopy (ICP-AES), UV-Vis Absorption Spectroscopy, and Raman Spectroscopy.

In this paper, comprehensive results demonstrating selective antioncogenic effects of novel green AgNPs acting selectively over cancer A375 cells was presented. We performed thorough characterization of the AgNPs derived from two synthetic approaches: colloidal and green synthesis. The analysis confirmed their nearly spherical morphology and comparable nanometric size, as shown by TEM acquisitions and DLS measurements, while also verifying their stability in cell culture media. Additionally, UV-Visible absorption spectra confirmed the expected absorption peaks. Raman spectroscopy further revealed distinct signals attributed to citrate groups for colloidal AgNPs, and to polyphenols aromatic rings for green AgNPs. Undoubtedly, the eco-friendly method leverages natural resources, such as plant extracts, to produce AgNPs and provide an effective and sustainable alternative for therapeutic and technological advancements. Based on this, the research aims to reveal pivotal molecular alterations with potential therapeutic implications.

The cell viability assay revealed that green AgNPs selectively reduced viability in A375 melanoma cells, while this effect was significantly less pronounced in the healthy HaCaT cell model.

The selectivity of green AgNPs over colloidal AgNPs in melanoma treatment likely stems from their unique synthesis process, which results in NPs with different physicochemical properties. As previously demonstrated, green AgNPs could interact with biological systems selectively. The green synthesis process in fact eliminates toxic chemicals, allowing for a safer and more targeted action on cancer cells. This selective inhibition could be attributed to the difference in surface chemistry, size, and reactivity between the two types of NPs [34].

Notably, this selective antitumor effect was not observed when A375 cells were treated with colloidal AgNPs. Hence, we performed a proteomic DIA/SWATH screen to reveal the molecular background of the shift in A375 proteotypes induced by green AgNPs treatment in two timepoints and benchmarked it to colloidal AgNPs treatment. Strikingly, the phenotypic changes and global proteome changes were reflected even on molecular level, mechanistically elucidating the antioncogenic effect of green AgNPs on A375 melanoma cells.

Initially, we defined the difference in molecular effects on A375 cells after exposure to colloidal AgNPs and green AgNPs. Partially in line with cell viability assay, colloidal AgNPs acted less effectively over the A375 cancer cells. The 24 h colloidal AgNPs treatment led to the dysregulation of only two proteins. Even prolonged exposure to colloidal AgNPs did not provide an extensive effect. A relatively small set of dysregulated proteins, characteristic by downregulated BIP, S38A2, and ASNS, and upregulated MCM4, CNN3, DNJA1, PP1G, TYSY, UHRF1, and UBE2T, with a prevalence of nuclear expression, was detected. The downregulated subset of proteins contains proteins involved in transmembrane transport, an endoplasmic reticulum chaperone system [35], regulator of the mitotic cell cycle [36], and programmed cell death [37].

Conversely, the small subset of upregulated proteins harbors proteins involved in cell differentiation and signaling processes, a translation regulator, chromatin organization [38], DNA replication [39] and repair, the mitotic cell cycle, and cell adhesion-mediated activity [40]. At first glance, it appears that the effects of colloidal AgNPs on A375 cells involve multiple processes, including general nanoparticle-induced cellular responses as well as pathways associated with cancer. However, the set of affected proteins is too limited to confidently conclude that the impact of colloidal AgNPs is truly antitumorigenic. However, after inspecting the dysregulated protein pool that occurred in response to green AgNPs at 24 h, compared to nontreated cells, we see a much greater response with a much more dysregulated protein pool. The protein pool includes SP1, CCD9B, NOL3, DPOA2, RM33, DSG2, YMEL1, ZN740, and many others, predominantly downregulated at 24 h. Multiple proteins are crucial for cellular proliferation, survival, and differentiation. This reflects the cellular response primarily to DNA damage, programmed cell death, and autophagy. A deeper analysis of the downregulated protein pool following 24 h of green AgNP treatment reveals the modulation of mechanisms related to mitochondrial organization, electron transport within the respiratory chain, signaling pathways, and cell junction organization. These processes are not only associated with the cellular response to nanoparticle exposure but also play crucial roles in cellular metabolism and cancer progression. Notably, the impact of green AgNPs on A375 cells becomes even more pronounced after 48 h of treatment.

There is a notable prevalence of altered protein expression, both in the nucleus and cytoplasm, along with a remarkable presence of proteins localized to the endoplasmic reticulum. The expression profiles of several targeted proteins exhibit both downregulation and upregulation. Notably, proteins displaying increased levels along with treatment, such as TPP1, VTDB, PCKGM, SP1, ALDH2, and LEG3, play crucial roles in various biological processes, such as mitochondrial function, vitamin D transport, gene expression modulation, and inflammatory responses. Among these, ALDH2 and PCKGM are mitochondrial enzymes, while VTDB is a secreted factor involved in vitamin D transport, scavenging extracellular G-actin, and enhancing chemotactic activity [41]. SP1 functions as a transcription factor, while LEG3 acts as a pre-mRNA splicing factor in the nucleus and is implicated in inflammatory responses [42], cell adhesion, and cell differentiation [43].

Notably, MT1X and MT2, which are metallothioneins known for their involvement in the detoxification process due to their metal ion binding function, exhibited upregulation over the course of green AgNP treatment. Interestingly, the existing literature describes their upregulation in melanoma disease [44]. Conversely, several proteins exhibit downregulation over the course of the treatment in cellular processes, each contributing to distinct functions within the cell, including protein synthesis, calcium signaling and cell motility, mitochondrial protein transport and stress response, DNA repair, autophagy, cell cycle regulation and chromatin organization, and DNA stability regulation, which might also greatly contribute to the apoptosis of A375 cells. Clearly, these findings pave the way for further exploration of their functional implications in physiological and pathological processes.

Overall, these findings emphasize the intricate interplay of proteins in maintaining cellular homeostasis and underscore the importance of further exploration into their roles in both physiological and pathological contexts. Consequently, we conducted a reanalysis of our proteomic data, specifically focusing on the regulatory shifts in known oncogenes as influenced by the treatment. Additionally, we focused on protein networks/pathways that are linked by GO terms that are providing cumulative evidence of the green AgNPs treatment effect. Strikingly, within just 24 h of green AgNP treatment, we identified a significant early downregulation of several oncoproteins, with this effect intensifying further after 48 h. In the following sections, the roles of these suppressed oncogenes in the context of cancer-related cellular signaling pathways were explored.

We hypothesize that the suppression of these oncoproteins may inhibit pathways critical for cancer signaling, collectively resulting in the arrest of cancer growth through the action of the nanoparticles. In particular, DSG2, which is required to maintain the epithelial stability acting on cell-to-cell adhesion, targets PI3K/AKT, Hedgehog (Hh), and Wnt/β-catenin, which are widely implicated in human cancer and cancer cell models [45], as well as Sp1, a complex key transcription factor that acts on essential oncogenes, tumor suppressors, and housekeeping genes critical for the hallmarks of cancer. DSG2 also promotes tumor-associated angiogenesis through pro- and anti-angiogenic genes, allowing replicative immortality activity through p53 and Telomerase activity [46]. In addition, YME1L is required for the mitochondrial regulation of morphology, function, and plasticity, and also exerts an antiapoptotic activity and promotes oxidative stress [47]. Several studies have linked Ca^2+^ signaling disorders with carcinogenesis and tumor progression [48]. Among the plot outcomes, TMCO1, a calcium-selective channel, can prevent excessive calcium storage, thus contributing to calcium homeostasis. Calcium-related pathways are involved both in melanogenesis and in melanoma tumorigenesis, in which the calcium influx specifically, across multiple cell compartments in a primary melanoma, represents a key regulator of the whole process [49]. Strikingly, the Wnt signaling pathway might have been impacted by the influence of nanoparticles treatment by GIPC2, a member of the GIPC family of proteins, which is known to activate the Wnt routes. In [50], the expression levels of GIPC2 were analyzed in a panel of cancers, with results indicating that the levels were mainly low in cancer types compared with the tissue adjacent to the cancer. CDK4 acts as “master regulator” of the cell cycle, and it has been proven that many human tumors share a CDK4/6-CYCLIN D-INK4-RB pathway altered by multiple mechanisms [51]. The Cyclin D-CDK4 specifically contributes to various mitogenic and antimitogenic signals. In this context, PDS5B was also influenced by the treatment, and as regulator of the cohesion of sister chromatid in mitosis, it affects the cohesion and repair processes of DNA; it has a proven role in carcinogenesis [52] and progression in prostate cells. Lastly, other reported signals refer to HMGB1, a pro-inflammatory mediator responsible for tumor formation, progression, and metastasis in many cancers [53]; UHRF1 has a DNA damage sensor activity [54] and is highly expressed in many human cancers compared to normal tissues, as well as being also associated with rapid disease progression [55]. Among the oncogenes shown, DNAJA1 exerts a role in the prevention of unfolded mutant p53 from proteasomal degradation, although its biological role remains unknown [56]. In summary, we conclude that the observed selective decrease in cancer cell viability is likely driven by the inhibition of the oncogenes discussed. The identified pool of suppressed oncoproteins sheds light on the mechanism underlying the selective action of green AgNPs, which effectively halts cancer growth—a phenomenon not observed with colloidal AgNPs.

Moreover, gene ontology revealed a significant shift in metabolism of carboxylic acids underlaid by upregulation of almost 17 proteins knowingly involved in this process (GO). Some clinically significant hallmarks of cancer may offer additional insights into the upregulation of this process during green AgNP treatment, particularly aerobic glycolysis, which is a metabolic adaptation supporting rapid proliferation, robust anabolism, evasion of apoptosis, and metastatic potential [57]. This biochemical shift may further underscore the restorative effects of the tested green AgNPs, as the observed increase in carboxylic acid metabolism suggests enhanced Krebs cycle activity, a metabolic characteristic more typical of normal cells. In this panel (GO: 0019752 Carboxylic acid metabolic process term) not all 17 highlighted proteins link to the Krebs cycle directly; however, there are several proteins whose increased activity have more direct links, such as GLUD1, MCCC2, TIGAR, HSD17B4, and PYCR2. TIGAR inhibits glycolysis by reducing fructose-2,6-bisphosphate in a p53/TP53-dependent manner, leading to the activation of the pentose phosphate pathway (PPP) and promoting NADPH production [58,59]. It also lowers ROS levels by generating reduced glutathione, thereby protecting cells from oxidative and metabolic stress [59]. Additionally, TIGAR supports cancer cell survival by activating DNA repair through PPP in a CDK5-ATM-dependent signaling pathway, in response to hypoxia or DNA damage [60]. Then PCKGM, a mitochondrial phosphoenolpyruvate carboxykinase, plays a crucial role in gluconeogenesis and the cellular response to glucose and tumor necrosis factor. Studies have shown that elevated expression of cytosolic phosphoenolpyruvate carboxykinase is linked to tumor repopulation in melanoma cells [61]. In summary, our reanalysis of quantitative proteomic data revealed a dysregulation of oncogenes and cancer-related metabolic processes. The shift in protein expression induced by the treatment favors an antitumorigenic effect, leading to the selective elimination of cancer cells. Notably, this is accompanied by a reduction in oncogene levels and a restoration of normal glucose metabolism, a selective effect of green AgNPs that was not observed with colloidal AgNPs.

## 4. Materials and Methods

### 4.1. Synthesis of AgNPs

AgNPs were synthesized using an eco-friendly approach outlined in [33]. Leaves of *Laurus nobilis* were gathered, cleansed with MilliQ water to eliminate impurities, and air-dried at ambient temperature for 24 h. Subsequently, 10 g of these leaves were immersed in a glass flask containing 100 mL of MilliQ water (in a 1:10 ratio) and boiled at 100 °C for 20 min. After cooling, the extract was filtered to prepare it for nanoparticle synthesis. Five mL of the purified extract was added to a solution containing AgNO_3_ dissolved in MilliQ water. The mixture was stirred at 400 rpm for 1 h at room temperature until the color of the solution turned from yellow to brown. Upon this color change, the solution underwent centrifugation at 4000 rpm for 1 h to gather the nanoparticles in the pellet, which were then purified with MilliQ water through three cycles of centrifugation at 13,000 rpm.

### 4.2. Nanoparticles Characterization

The shape and spread of the magnetic nanoparticles (MNPs) were analyzed using a JEOL JEM-1011 transmission electron microscope (TEM, Jeol LTD., Tokyo, Japan) running at 100 kV. To prepare the nanoparticles, a few microliters of the nanoparticle solution were dropped onto a copper grid coated with formvar. The images of the free MNPs presented were taken with grids that were prepared immediately after the synthesis process, using a few microliters of highly concentrated final suspensions diluted in an appropriate buffer or water, depending on the surface coating. For DLS and ζ-potential measurements of AgNPs in aqueous solutions (at 25 °C and pH 7), a Zetasizer Nano-ZS equipped with a HeNe laser (633 nm, 4.0 mW) and a detector (ZEN3600, Malvern Instruments Ltd., Malvern, UK) was employed.

UV-vis analysis spanning the spectral range of 300 ÷ 800 nm was conducted at room temperature using a Varian Cary 5 spectrophotometer (ZEN3600, Malvern Instruments Ltd., Malvern, UK) equipped with a quartz cuvette that had a 10 mm path length.

The Raman spectra of Ag-NPs and Green Ag-NPs were obtained using a MicroRaman Xplora (Horiba, Bhamboli, Maharashtra, India) with a laser source at 532 nm.

### 4.3. Determination of AgNPs Concentration

The concentrations of colloidal AgNPs and green AgNPs were determined through elemental analyses employing ICP-OES Perkin Elmer AVIO 500. In this process, 150 μL of the NP solutions were digested overnight by adding 1.5 mL of HNO_3_, followed by dilution with MilliQ water (at a 1:10 ratio).

### 4.4. Cell Culture

Human melanoma cells (A375) and human epidermal keratinocyte line (HaCaT) were maintained in Dulbecco’s Modified Eagle Medium (DMEM) (Sigma-Aldrich, Dorset, UK) enriched with 2 mM glutamine, penicillin/streptomycin (Sigma-Aldrich, Dorset, UK) and 15% of Fetal Bovine Serum (FBS) (Sigma-Aldrich, Dorset, UK). Cells were grown in a humidified controlled atmosphere with a 95 to 5% ratio of air/CO_2_, at 37 °C, and to 97% confluence prior to treatment with nanoparticles.

A375 and HaCaT cells were seeded starting from a 97% of confluence, with 5.27 × 10^5^ cells for 1 mL; thus, an initial number of cells in 7 mL (3.689 × 10^6^) were counted using a T20 Automated cell counter (Bio-Rad, Hercules, CA, USA).

In each well, we seeded 400 µL of cells in 1.6 mL DMEM, thus having 1.85 × 10^6^ cells at a concentration of 5 × 10^3^ cells per well in 6-well plates and stabilized in a humidified controlled atmosphere with a 95 to 5% ratio of air/CO_2_, at 37 °C for 24 h.

### 4.5. Cell Viability Assessment and Uptake

A375 and HaCaT were seeded at a concentration of 5 × 10^3^ cells per well in 96-well plates and stabilized in a humidified controlled atmosphere with a 95 to 5% ratio of air/CO_2,_ at 37 °C for 24 h. Subsequently, green and colloidal AgNPs at three different concentrations (1 µM, 3 µM, and 5 µM) were added to cell media. After incubation times of 24 and 48 h, a standard WST-8 assay (96992, Sigma Aldrich, Dorset, UK) was used. WST-8 is a water-soluble tetrazolium salt (WST) [2-(2-methoxy-4-nitrophenyl)-3-(4-nitrophenyl)-5-(2,4-disulfophenyl)-2H-tetrazolium. The control tests were performed on cells incubated with equivalent volumes grown solution of 10% (*v*/*v*) dimethyl sulfoxide (DMSO, Sigma-Aldrich, Dorset, UK) in Dulbecco’s phosphate-buffered saline (DPBS). Samples were measured using a Fluo Star Optima (BMG LABTECH, Ortenberg, Germany) microplate reader at a wavelength of 460 nm. Values were collected and elaborated with MARS Data Analysis Software (BMG LABTECH), OPTIMA series V3.01 R220.12.2013. The data, obtained on 8 different viability experiments, were expressed as mean ± SD. The concentrations of the internalized colloidal and green AgNPs were calculated by elemental analyses using an Avio^®^ ICP-OES 500 (PerkinElmer Inc. Waltham, MA, USA). To estimate the intracellular NPs uptake, 10^5^ cells were seeded in 1 mL of medium in each well (3.5 cm diameter) of a 6-well plate. After 24 h, the medium was replaced with fresh medium containing the AgNPs (colloidal and green) at a concentration of 5 µM for 48 h. After this time, the medium was removed, and the cells were washed three times with PBS (pH 7.4) to remove non-internalized, membrane-bound NPs. Then, cells were trypsinized and counted using a cell-counting chamber. Next, the cell suspensions were digested using 1 mL of concentrated HNO_3_ 10% (*v*/*v*), and the intracellular Ag concentration was measured by elemental analysis. The Ag content was measured in 360,000 cells (ng Ag) for the two cell lines.

### 4.6. Sample Preparation for Mass Spectrometry

Human melanoma cells (A375) and human epidermal keratinocyte line (HaCaT) were grown as described in Section 4.4. The label free data independent acquisition (DIA) quantitative proteomics method was employed to study the proteotype changes triggered by AgNPs. A375 melanoma cancer cells were exposed to 3 μM colloidal and green AgNPs for 24 and 48 h, and subsequently, their impact on the differential regulation of proteins was studied. Control samples (WT cells) consisted of A375 melanoma cells in DMEM medium with no nanoparticle treatment. Other than the control samples, all the timepoints and treatments were prepared in three discrete wells in a 6-well cell culture plate (Sarstedt, Germany) and grown up to 97% cell confluency yielding three biological repetitions. Next, cultured cells were washed three times on the cell culture plate with PBS (phosphate buffered saline) and scraped into the lysis buffer consisting of 8 M Urea in 0.1 M Tris/HCl, pH 8.5 (Urea buffer). Samples were then snap frozen in liquid nitrogen two times, sonicated in the sonication bath (ELMA, Pforzheim, Germany) for 5 min, and left on ice for 30 min. Protein lysates were cleared by centrifugation at 30 min/14,000× *g* at 8 °C. Clean supernatants were transferred into the clean low binding tubes. The protein concentration in the sample was determined by BCA protein assay according to the manufacturer’s recommendations (Thermo, Waltham, MA, USA). Approximately 50 µg of protein from each sample was taken for digestion using a modified filter-aided sample preparation protocol (FASP) [62].

Microcon 10 kDa cut-off filter units (Millipore, Burlington, MA, USA) were used for tryptic digestion. Briefly, 200 μL of Urea buffer and a volume of lysate corresponding to 50 μg of protein were added to the filter unit. Filter units were centrifuged at 17,000× *g* at 20 °C for 30 min. For protein reduction, 100 μL of Urea buffer and 20 μL of 100 mM Tris (2-carboxyethyl) phosphine hydrochloride (Sigma-Aldrich, St. Louis, MO, USA) were added to the filter unit, and the mixture was incubated for 30 min on a thermoblock set at 37 °C/600 rpm. The filter unit was then centrifuged at 17,000× *g* for 20 min. Protein alkylation was initiated by adding 20 μL of 300 mM iodoacetamide (Sigma-Aldrich, St. Louis, MO, USA) in 100 μL of Urea buffer and incubated in the darkness for 20 min at room temperature, followed by centrifugation at 17,000× *g* for 20 min. Next, filter units were washed three times with 100 μL of 100 mM NH_4_HCO_3_, then centrifuged at 17,000× *g* for 30 min; the step was repeated three times in total. After changing collection tubes, 100 μL of 50mM NH_4_HCO_3_ and 1.5 μL of 0.5 μg/μL trypsin solution in ddH_2_O (Promega, Madison, WI, USA), were added for the overnight digestion at 37 °C. Tryptic peptides were collected the next day by centrifugation at 17,000× *g* for 15 min. Filter units were washed with an additional 50 μL of 0.5 M NaCl in LC-MS water, through spin down at 17,000× *g*/15 min.

Then, the peptide desalting on MicroSpin C18 columns (Harvard Apparatus, Holliston, MA, USA) was performed via a modified protocol by Bouchal et al. [63]. The presence of excessive salts interferes with peptide ionization and contributes to the compromised mass spectrometry analysis. Briefly, MicroSpin C18 columns (Harvard Apparatus, Holliston, MA, USA) were twice conditioned with 200 μL of acetonitrile (ACN) + 0.1% formic acid (FA) while trying to avoid any bubbles formation on the frit surface, and subsequent spinning down at 200 rpm/2 min. The step was repeated 2 more times. Next, column equilibration required 200 μL of ddH_2_O + 0.1% FA followed by a centrifugation step at 300 rpm/2 min. The step was repeated two times in total. The C18 slurry was then hydrated by adding 200 μL of ddH_2_O + 0.1% FA, leaving it hydrating for 15 min followed by centrifugation at 300 rpm/2 min. Tryptic peptides were then transferred onto the activated C18 column and centrifuged at 500 rpm/2 min. Following desalting was repeated 3 times in total by adding 200 μL of ddH_2_O + 0.1% FA, followed by a centrifugation at 500 rpm/2 min. Afterwards, C18 columns were transferred to a new low binding collection tube, and peptides were first eluted by 200 μL of 50% ACN + 0.1% FA, then centrifuged at 500 rpm/2 min. Next, elution steps were done two times by adding 200 μL of 80% ACN + 0.1% FA each time, followed by centrifugation at 500 rpm/2 min. Desalted peptide eluates were dried by vacuum centrifugation for 3 h at 30 °C in a speedvac concentrator (Eppendorf, Hamburg, Germany). Dry samples were stored at −80 °C until the LC-MS analysis.

### 4.7. Liquid Chromatography-Mass Spectrometry (LC-MS/MS) Analysis

Liquid chromatography and mass spectrometry were performed based on a method described in our previous publication. Briefly, samples were dissolved in 50 μL of 0.08% trifluoroacetic acid (TFA), 2.5% ACN in LC-MS water, and approximately 1 μg of peptides were loaded into the fluidic system of liquid chromatograph UltiMate™ 3000 RSLC nano System (Thermo Scientific, Waltham, MA, USA). Identical liquid chromatography conditions were used for both data dependent (DDA) and data independent (DIA) acquisition. Peptides were trapped on Acclaim PepMap 100, 5 µm particle size, 1 mm inner diameter, 5 mm length C18 pre-column (cat. no: 16045, Thermo Scientific, Waltham, MA, USA). Loading pump flow was kept at 5 µL/min with an isocratic mobile phase composition of 0.08% TFA, 2.5% ACN in LC-MS water. Analytical peptide separation was done on an analytical PepMap™ 100, 2 µm particle size, 1 mm inner diameter, 5 mm length C18 analytical column (cat.no: 164534, Thermo Scientific, Waltham, MA, USA). Peptides were separated by a linear gradient of 0.1% FA in ACN (*v*/*v*) (B) and 0.1% FA in water (*v*/*v*) (A). Analytical peptide separation was started at 2.5% B, followed by increasing its proportion up to 40% B in A over 90 min. A column flush at 95% B was performed over the next 8 min, and the final 8 min were left for column equilibration at 2.5% B. Nano-electrospray was used to ionize the peptides, followed by their introduction into the Orbitrap Exploris™ 480 mass spectrometer (Thermo Scientific, Waltham, MA, USA).

One technical replicate was acquired by a DDA method. The resolution for full scan operated in a profile mode was set to 120,000. Full scan scanned through a mass range spanning from *m*/*z* 350 Th up to *m*/*z* 1200 Th. Normalized AGC target was set to 300% with the auto setting on maximum injection time. MS/MS scans were performed on the top 15 most intense precursor ions selected from the full scan. Once fragmented precursor ions were excluded for 20 s with precursor exclusion mass tolerance set to 10 ppm, minimum precursors with ion intensity below 5.0 × 10^3^, and precursors outside of charge states range of +2 to +6 were suspended from the experiment. The precursor isolation window width was 2 Th. The collision energy was set to 30%, and the normalized collision energy type was selected. The MS/MS scan was operated in a centroid mode, and the resolution was set to 120,000. Standard setting for normalized AGC target and 40 ms maximum injection time was set.

### 4.8. Data Independent Acquisition

Two technical replicates of each biological replicate were measured. DIA acquisition was accompanied by a full scan at 60,000 resolutions, and a mass range from *m*/*z* 350 Th up to *m*/*z* 1450 Th was considered. The normalized AGC target was set to 300%, and maximum injection time was 100 s. The DIA method cycled in a precursor mass range spanning from *m*/*z* 350 Th up to *m*/*z* 1100 Th, which was divided into 12 Th wide windows with 1 Th overlap. Therefore, a single DIA cycle consisted of 62 MS/MS scans. For each MS/MS scan, a normalized collision energy type was set to 30%. Orbitrap resolution was set to 30,000. Normalized AGC target was set to 1000%, with an automatic setting on the maximum injection time parameter. The data type was profile.

### 4.9. Proteomics Data Analysis

Applying quantitative proteomics and data-independent acquisition (DIA) methodology enables the evaluation of the proteome landscape after different types of AgNPs treatments on melanoma cancer cells in different timepoints. Quantitative analysis is an essential aspect of biomarker discovery, and therefore, we have performed a comparative bioinformatic evaluation of proteomic landscapes upon the colloidal and green AgNPs at 24- and 48-h timepoint treatments, as described below.

### 4.10. Spectral Library Generation and DIA Data Extraction

A MaxQuant search engine was implemented to search raw unprocessed DDA files. *Homo sapiens* SwissProt + UniProt search database (06_2023) and a reverse decoy database harboring an equal number of decoy sequences and common contaminant protein sequences were used to perform database search. Enzyme digestion was set to trypsin with allowance of two missed cleavages. Carbamidomethylation of cysteine was considered as a fixed modification. The list of variable modifications harbored methionine oxidation, protein N-term acetylation. Other processing settings were left as default. MaxQuant output files (MSMS file) were further used in Skyline-daily (64-bit, 20.1.9.234) software which transformed them into a spectral library .blib file. The “Score Threshold” was left as default at 0.05 MaxQuant PEPscore. The FASTA file used for search was set for Skyline target generation background proteome. Peptides from .blib file were associated with proteins in Skyline target panel. Protein intensity extraction was performed in Skyline-daily (64-bit, 20.1.9.234) with identical settings to those described in Faktor et al. [25]. Briefly, zero missed cleavages were allowed in the Peptide settings tab. Maximum peptide length was set from 2 to 60 amino acids, and modified peptides were suspended from experiment. The precursor ion in a charge state from +1 to +5 and y and b product ions with +1 and +2 charges were included in the experiment. Product ion higher than the ion 4 up to the last product ion in series were included in the experiment. Ion match tolerance was set to 0.05 *m*/*z*. Peptides defined by at least 4 product ions were kept in the experiment, while up to the 6 most intense product ions were selected for precursors where more product ions were available. The DIA isolation scheme was auto-imported from the raw DIA file. Orbitrap resolution was set to 30,000 at 200 *m*/*z*. Reversed decoy sequences were created from target sequences, which were subsequently used for training the mProphet peak scoring model.

### 4.11. Statistical Analysis of Skyline Extracted DIA Data

R (version 4.0.0) package MSstats 4.0.1. was used for statistical analysis of extracted intensities. mProphet q-value threshold was set to q value < 0.01. After summing the transition intensities, the resulting peptide intensities were log2 transformed and quantile normalized. Protein quantitation was performed pairwise via mixed-effect models implemented in the MS stats group Comparison function. T-test *p*-values were Benjamini Hochberg adjusted. The full protein comparison matrix is available in the dataset, as described in the data availability section.

### 4.12. Data Visualization

Data were preprocessed using Python 3.9 and RStudio 2023.06.01. Volcano plots were generated in Enhanced Volcano 1.10.0. Inkcape 1.2 was used to process the graphics to final figures and to generate svg images.

## 5. Conclusions

Green AgNPs synthesized through eco-friendly methods using plant extracts offer significant advantages due to their biocompatibility and reduced environmental impact. These nanoparticles retain the unique physicochemical properties of colloidal AgNPs, such as a high surface area-to-volume ratio, which enhances their interaction with biological systems and promotes efficient cellular uptake. Additionally, the optical and plasmonic properties of AgNPs, including localized surface plasmon resonance, make them highly effective as active therapeutic tools in cancer treatment. The green synthesis approach also eliminates the need for toxic chemicals, making these nanoparticles a safer option for therapeutic applications. Our study employs the DIA/SWATH-MS technique to generate comprehensive quantitative proteomic profiles, revealing proteome alterations in cancer cells in response to nanoparticle treatment over time. For years, researchers have been exploring AgNPs for medical applications and drug delivery, with both colloidal and green AgNPs tested on melanoma cells. Colloidal AgNP treatment has proven less effective against melanoma in both the short and long term. In contrast, green AgNPs demonstrate promising results, selectively inhibiting melanoma cell growth while being safer for normal cells in terms of cell viability. Green AgNP treatment, both short- and long-term, induces dysregulation of key proteins involved in three primary mechanisms: suppression of oncogene expression, increased carbohydrate metabolism, and disruption of copper homeostasis. Looking ahead, we believe our approach will serve as a valuable resource for researchers interested in using DIA/SWATH quantitative MS proteomic analysis to evaluate nanoparticle treatments. This analysis is essential for understanding their therapeutic potential, safety, biological mechanisms, and for biomarker discovery.

There is a clear and pressing need for further studies to identify specific protein targets for therapy and to explore the potential of green AgNPs, either alone or in combination with other drugs, in melanoma treatment. This DIA/SWATH quantitative MS proteomic study marks the first step toward demonstrating the direct application of green AgNPs in melanoma therapy, and we eagerly anticipate future studies that will build on this foundation. Our findings suggest that green AgNPs could offer a safer and more selective approach for melanoma treatment compared to conventional colloidal AgNPs. By minimizing toxicity to healthy cells while effectively targeting melanoma cells, these nanoparticles may act as a foundation for future nanomedicine-based therapies. While our study provides a proteomic perspective on their effects, additional research, particularly in vivo studies and clinical trials, will be essential to validate their therapeutic potential.

## Figures and Tables

**Table 1:** DLS and zeta potential values of green and colloidal AgNPs in water and DMEM.

	**DLS in Water**	**Zeta Potential**
Colloidal AgNPs	21 ± 3 nm	−23 ± 2 mV
Green AgNPs	36 ± 5 nm	−30 ± 6 mV
	**DLS in DMEM**	**Zeta Potential in DMEM**
Colloidal AgNPs	28 ± 3 nm	−32 ± 5 mV
Green AgNPs	38 ± 7 nm	−41 ± 6 mV

**Table 2:** The accumulation in A375 and HaCat cells exposed to 5 µM for 48 h of colloidal and green AgNPs. Data were statistically significant for *p* < 0.05.

	A375	HaCaT
ng [Ag] from colloidal AgNPs	3.8 ± 3	4.8 ± 2
ng [Ag] from green AgNPs	4 ± 1	5.2 ± 3

**Table 3:** The list of the proteins from the three primary mechanisms have dysregulations in the melanoma cells to the treatment of green AgNPs.

Group	Protein	Gene	Comparison	log2FC	Adj.pvalue	Fold Change
**Oncogene**	DNJA1	DNAJA1	GrAg24_to_GrAg48	0.52	0.015	1.44
	CDK4	CDK4	GrAg24_to_GrAg48	0.96	0.032	1.94
	PDS5B	PDS5B	GrAg24_to_GrAg48	1.36	0.037	2.57
	HMGB1	HMGB1	GrAg24_to_GrAg48	1.39	2.82 × 10^−4^	2.61
	UHRF1	UHRF1	GrAg24_to_GrAg48	1.4	0.015	2.64
	TMCO1	TMCO1	GrAg24_to_GrAg48	1.41	0.035	2.66
	GIPC2	GIPC2	GrAg24_to_GrAg48	1.49	0.039	2.81
	CD123	CDC123	GrAg24_to_GrAg48	1.94	0.029	3.83
	PTBP3	PTBP3	GrAg24_to_WT	−1.6	0.018	0.33
	SP1	SP1	GrAg24_to_WT	−2.4	4.28 × 10^−4^	0.19
	DSG2	DSG2	GrAg24_to_WT	−2.91	0.018	0.13
	YMEL1	YME1L1	GrAg24_to_WT	−3.77	0.025	0.073
**GO:0019752 Carboxylic acid metabolic process**	SYYC	YARS1	GrAg24_to_GrAg48	−0.41	0.013	0.75
	ASNS	ASNS	GrAg24_to_GrAg48	−0.78	0.015	0.58
	SYCC	CARS1	GrAg24_to_GrAg48	−0.70	0.015	0.61
	GFPT1	GFPT1	GrAg24_to_GrAg48	−0.92	0.015	0.53
	ENOPH	ENOPH1	GrAg24_to_GrAg48	−0.94	0.015	0.52
	P5CR2	PYCR2	GrAg24_to_GrAg48	−0.68	0.027	0.63
	OSBL1	OSBPL1A	GrAg24_to_GrAg48	−0.49	0.015	0.71
	SYAC	AARS1	GrAg24_to_GrAg48	−0.43	0.033	0.74
	ACY1	ACY1	GrAg24_to_GrAg48	−0.77	0.032	0.59
	SYEP	EPRS1	GrAg24_to_GrAg48	−0.34	0.032	0.79
	GLYC	SHMT1	GrAg24_to_GrAg48	−0.90	0.033	0.54
	UGDH	UGDH	GrAg24_to_GrAg48	−0.60	0.037	0.66
	DHE3	GLUD1	GrAg24_to_GrAg48	−0.39	0.037	0.76
	MCCB	MCCC2	GrAg24_to_GrAg48	−0.42	0.048	0.75
	TIGAR	TIGAR	GrAg24_to_GrAg48	−0.51	0.048	0.70
	DHB4	HSD17B4	GrAg24_to_GrAg48	−0.53	0.037	0.69
	AK1C3	AKR1C3	GrAg24_to_GrAg48	−0.90	0.048	0.53
	PCKGM	PCK2	GrAg24_to_GrAg48	−2.15	0.027	0.22
**Copper homeostasis**	MT2	MT2A	GrAg24_to_GrAg48	−1.75	1.62 × 10^−4^	0.30
	MT1X	MT1X	GrAg24_to_GrAg48	−3.79	1.62 × 10^−4^	0.073

## Data Availability

The data presented in this study are available in this article. Mass spectrometry data are available via ProteomeXchange [64] via the PRIDE [65,66] partner repository with identifier PXD058049.

## Supplementary Materials

The following supporting information can be downloaded at: https://www.mdpi.com/article/10.3390/ijms26052029/s1.
