# Supplementary figures and images for: DIA/SWATH-Mass Spectrometry Revealing Melanoma Cell Proteome Transformations with Silver Nanoparticles: An Innovative Comparative Study
Source: Int J Mol Sci. 2025 Feb 26;26(5):2029. doi: 10.3390/ijms26052029 (PMC11901134; doi:10.3390/ijms26052029)

# Supplementary Materials

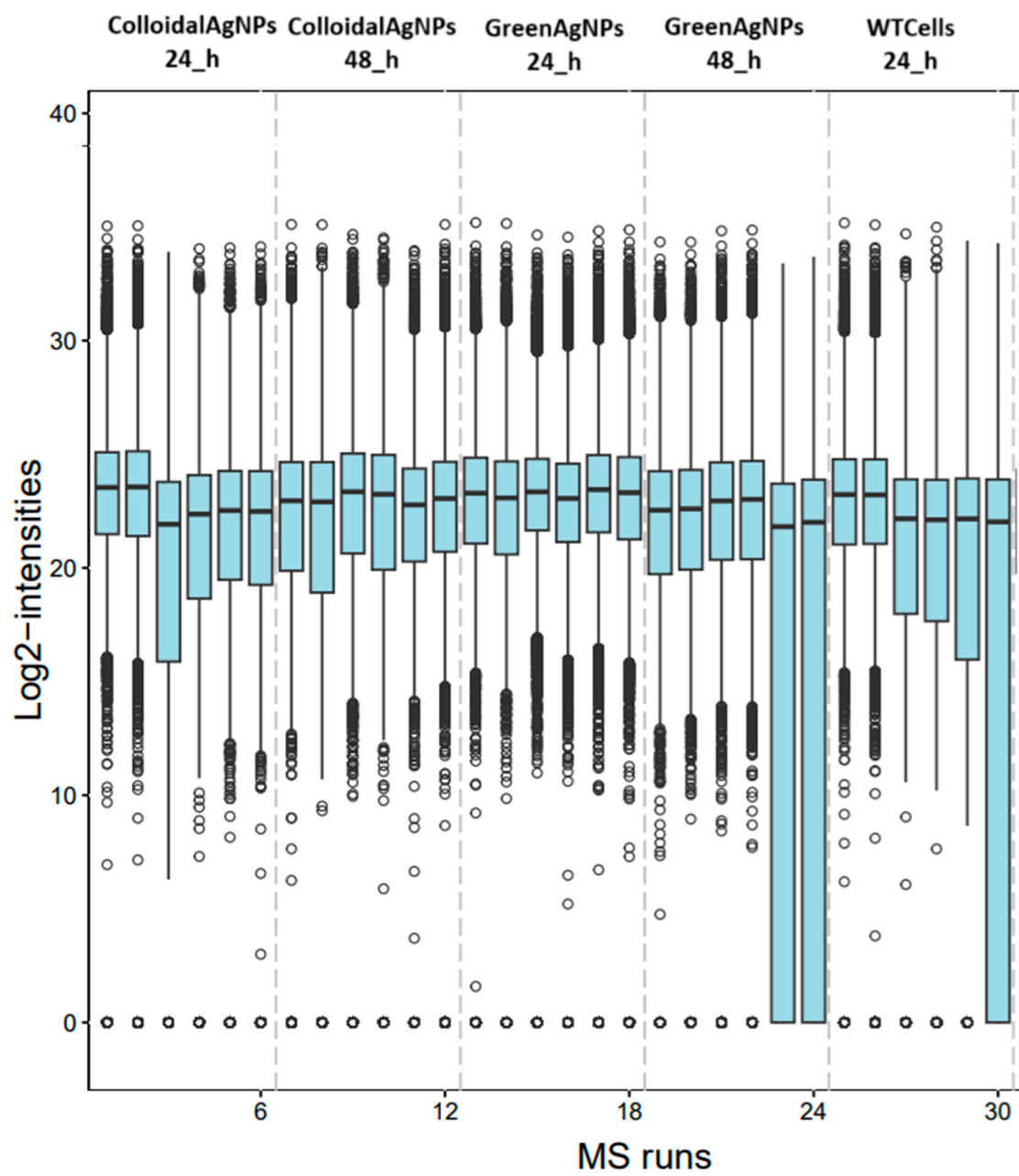

**Figure S1.** Protein intensity QC plot

Supplement: Supplementary file 1 [file ijms-26-02029-s001.zip › Supplementary Materials.pdf]
